# Magnetic measurements with atomic-plane resolution

**DOI:** 10.1038/ncomms12672

**Published:** 2016-08-31

**Authors:** Ján Rusz, Shunsuke Muto, Jakob Spiegelberg, Roman Adam, Kazuyoshi Tatsumi, Daniel E. Bürgler, Peter M. Oppeneer, Claus M. Schneider

**Affiliations:** 1 Department of Physics and Astronomy, Uppsala University, Box 516, S-75120 Uppsala, Sweden; 2 Advanced Measurement Technology Center, Institute of Materials and Systems for Sustainability, Nagoya University, Chikusa-ku, Nagoya 464-8603, Japan; 3 Peter Grünberg Institute, Electronic Properties (PGI-6), Forschungszentrum Jülich, 52425 Jülich, Germany

## Abstract

Rapid development of magnetic nanotechnologies calls for experimental techniques capable of providing magnetic information with subnanometre spatial resolution. Available probes of magnetism either detect only surface properties, such as spin-polarized scanning tunnelling microscopy, magnetic force microscopy or spin-polarized low-energy electron microscopy, or they are bulk probes with limited spatial resolution or quantitativeness, such as X-ray magnetic circular dichroism or classical electron magnetic circular dichroism (EMCD). Atomic resolution EMCD methods have been proposed, although not yet experimentally realized. Here, we demonstrate an EMCD technique with an atomic size electron probe utilizing a probe-corrected scanning transmission electron microscope in its standard operation mode. The crucial element of the method is a ramp in the phase of the electron beam wavefunction, introduced by a controlled beam displacement. We detect EMCD signals with atomic-plane resolution, thereby bringing near-atomic resolution magnetic circular dichroism spectroscopy to hundreds of laboratories worldwide.

## Abstract (web summary)

It has been predicted that electron beam probes may allow for the imaging of magnetism with atomic-scale resolution. Here, the authors demonstrate a scanning transmission electron microscopy method capable of resolving magnetic contrast from individual atomic planes.

## Graphical abstract

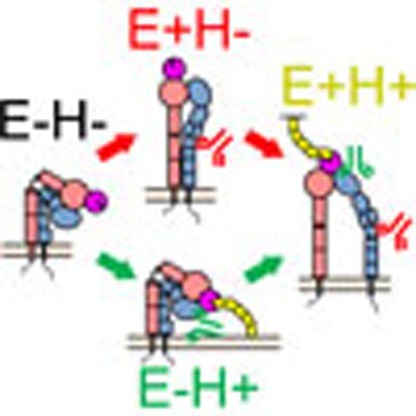

Magnetic circular dichroism in electron microscopy, electron magnetic circular dichroism (EMCD), was first observed in 2006 (ref. 1), following swiftly its prediction from 2003 (ref. 2). The technique attracted a lot of interest due to its analogy to X-ray magnetic circular dichroism^3^, as well as for its potential of reaching atomic resolution routinely available in probe-corrected scanning transmission electron microscopy (STEM). Since the inception of EMCD, a rapid development has followed, deepening the theoretical understanding^4^,^5^,^6^, initiating development of new experimental set-ups and improving the spatial resolution and quantitativeness^7^,^8^,^9^,^10^,^11^,^12^,^13^,^14^. However, the goal of achieving atomic resolution remains unaccomplished. The reasons are practical limitations of the classical EMCD technique combined with the low signal-to-noise ratios. In classical EMCD (ref. 1), the sample is tilted to a 2-beam or 3-beam orientation and signal acquisition is done on Thales circle positions aside the Bragg spots, greatly wasting electron counts. The resulting low-count rates then combine unfavourably with limited spectrum acquisition times in atomic resolution STEM, which are required to prevent sample damage or drifts. New inspiration came in 2010 when the generation of electron vortex beams was demonstrated^15^,^16^,^17^. As shown later by theory^18^,^19^, it should be possible to detect EMCD at atomic resolution with electron vortex beams having a non-zero orbital angular momentum. Notably, for this approach the atomic size of the probe is a requirement, not an option. Theoretical analysis has shown that the symmetry of the phase distribution in the wavefunction of the electron beam plays a crucial role^20^ in detection of EMCD. As a consequence, alternative beam shapes have been proposed for atomic resolution EMCD, such as astigmatic beams^20^. Yet, even today an EMCD detection with atomic size probes remains very demanding. The likely reasons are technical difficulties in generating atomic size electron vortex beams, together with fine control of their resulting orbital angular momentum in the case of electron vortex beams, and a low EMCD signal fraction in the case of astigmatic beams^22^.

In this work we demonstrate measurements of EMCD with atomic-plane resolution (APR-EMCD). Our approach combines ideas of both classical and atomic resolution EMCD. Theoretical formulation is supported by explicit simulations and experiments. The technique does not require any modifications of the microscope hardware and is thus accessible to hundreds of laboratories worldwide.

## Results

### Theory

**Figure 1:**
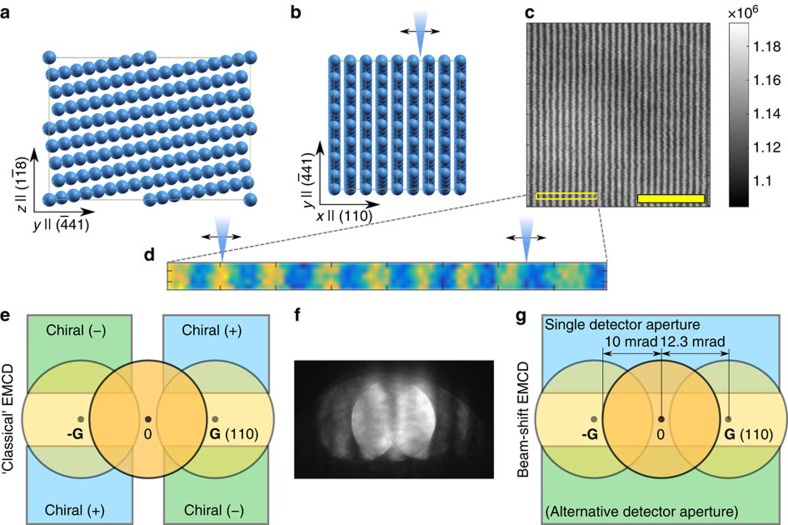
Scheme of the experimental set-up. (**a**) Side-view of the structure model of bcc iron tilted into a 3-beam orientation with the beam along the 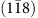 direction. (**b**) Top view of the structure model. Scanning direction is schematically shown by blue cone with arrows. (**c**) ADF survey image (acceleration voltage 200 kV, convergence semi-angle 10 mrad) showing atomic planes. The yellow scale bar denotes 2 Å. (**d**) ADF image acquired along with the acquisition of a spectrum image. Its size corresponds to the yellow rectangle marked in the ADF survey image. (**e**) Schematic placement of detectors for a classical EMCD approach in the 3-beam orientation. (**f**) Experimental diffraction pattern acquired in the same settings as the ADF survey image. (**g**) Detector placement in the here-proposed APR-EMCD approach. Note that in the classical approach we need two measurements at each beam position to acquire both chiral (+) and chiral (−) spectra, while in the APR-EMCD a single detector aperture is sufficient.

The APR-EMCD combines ideas of both classical and atomic resolution EMCD. From classical EMCD we borrow the concept of tilting the sample into a 3-beam orientation, see Fig. 1a. The advantage is a simplification of the dynamical diffraction effects, compared with zone-axis orientation. The price paid is that we lose the view of individual atomic columns, instead we can observe atomic planes as parallel lines in a high-angle annular dark field (ADF) STEM image, Fig. 1b,c. From the atomic resolution EMCD approaches we borrow the concept of manipulating the phase distribution in the probe wavefunction. Naturally this requires to use large convergence angles, leading to a coherent overlap of the discs of the diffracted beams. Following ref. 20, for a magnetization **M**=(0, 0, *M*) parallel to the beam direction and Bragg spots **G**_±_=±(*G*, 0, 0) along the *x* axis, the EMCD signals originate primarily from mixed dynamical form-factors^22^ proportional to the *y*-component of the momentum transfer *q*_y_ (see Methods section for a detailed derivation). These terms are multiplied by the sines of the phase difference Δ*ϕ*_**k,G**_=*ϕ*_**k**+**G**_−*ϕ*_**k**_. A plain shifting of the beam introduces a phase ramp 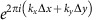 in the Fourier transformed wavefunction, as a trivial consequence of the Fourier shift theorem. Considering that the Bragg spots lie along the *x* axis in our reference frame, only the shift in *x*-direction matters, resulting in a phase difference of Δ*ϕ*_**k,G**_=2*πG*Δ*x* independent of *k*_*x*_, *k*_*y*_. The sine of such phase differences is maximized/minimzed when the beam shift Δ*x* equals 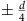, where *d* is the atomic-plane spacing. In other words, in the 3-beam orientation we expect to detect positive and negative EMCD signals in the neighbourhood of the atomic planes. The proportionality of the magnetic signal to *q*_y_ means that the detection can be done in the upper or lower diffraction half-plane. A scheme of the experimental set-up and the comparison to a classical EMCD experiment in 3-beam orientation is shown in Fig. 1d.

Let us discuss briefly the importance of this claim. It is proposed that a standard spectrum image measured on a sample tilted into a 3-beam orientation will contain EMCD signals next to the atomic planes. Hence, there is no need of beam shaping with angular momentum or aberrations, because the desired phase differences arise naturally as a consequence of shifting the beam during the scan. In addition, the method provides areas of both positive and negative EMCD in one and the same data set, acquired under identical measurement conditions, eliminating the need to scan the same area twice—once with positive and once with negative angular momentum or astigmatism. Consequently, the proposed APR-EMCD method sidesteps the technical issues associated with atomic resolution EMCD.

### Simulations

**Figure 2:**
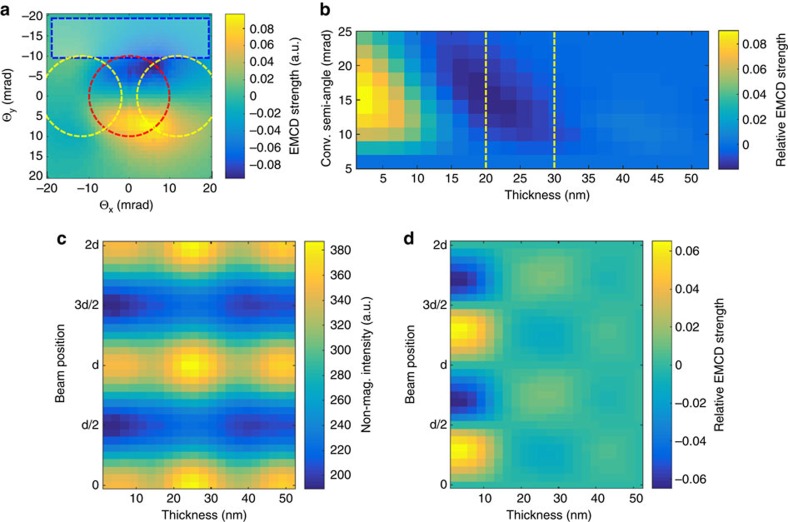
Simulations of EMCD in the 3-beam geometry. (**a**) Example distribution of the Fe-*L*_3_ edge EMCD signal in the diffraction plane for a beam positioned at 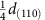 to the side from atomic planes. Positions of the transmitted beam disk (red circle) and Bragg scattered beam disks (yellow circles) are marked, as well as the rectangular detector region used in (**b**) the optimization of the relative EMCD signal strength as a function of convergence angle and sample thickness. (**c**) Calculated non-magnetic signal as a function of probe position and sample thickness at the Fe-*L*_3_ edge and (**d**) the corresponding relative EMCD strength. In **b**,**c** the convergence semi-angle was set to 10 mrad.

First we probe the validity of the APR-EMCD method by an explicit simulation of dynamical diffraction effects and inelastic scattering of an atomic size probe^23^ on an iron crystal tilted to a 3-beam orientation with **G**=(110) and the beam parallel to the (1
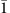
8) direction, which represents a tilt of 10° from the (001) crystal orientation. Figure 2 summarizes the results. An example distribution of the EMCD signal in the diffraction pattern for a beam shifted to the side of an atomic plane is shown in Fig. 2a. Note how this distribution differs qualitatively from the classical 3-beam EMCD, which displays EMCD of alternating signs in the four quadrants. See Supplementary Fig. 1 for an example of such a diffraction pattern. The rectangular detector was positioned as shown in Fig. 2a and a wide range of sample thicknesses and convergence angles has been evaluated. The resulting relative EMCD strength is shown in Fig. 2b, from which we conclude that an optimal convergence semi-angle for 20 and 30 nm-thick samples is 15 and 10 mrad, respectively. The latter conditions were then used to simulate the thickness and probe-position dependence of the non-magnetic signal, shown in Fig. 2c, and the corresponding relative strength of APR-EMCD signal, Fig. 2d, confirming the localization of the APR-EMCD signal at positions 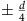 distant from the lattice planes.

### Experiments

**Figure 3:**
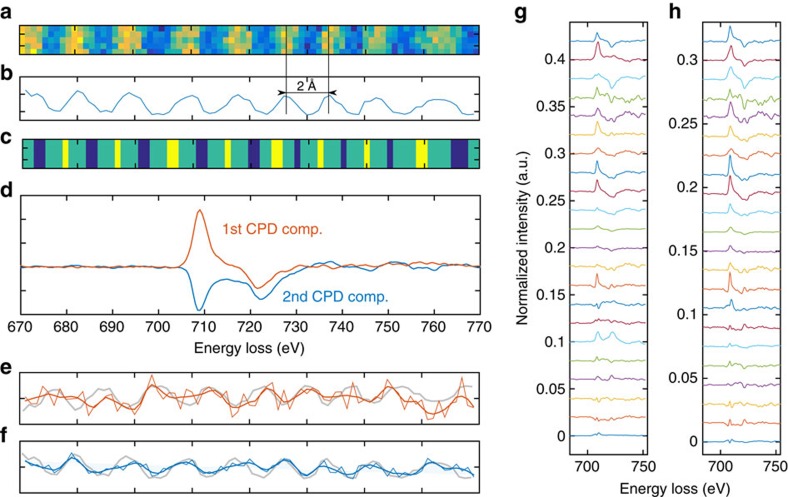
Experimental detection of APR-EMCD. (**a**) Representative ADF image of the scanned area and (**b**) its line profile. A smoothed profile was used to identify individual minima and maxima, based on which a mask has been defined (**c**) with blue and yellow regions marking summation areas centred at *d*/4 to the right or left of the atomic columns. Their width is determined by local steepness of the ADF line profile. (**d**) Example of the two CPD components with line profiles of their maps. (**e**,**f**) Note the strong correlation between the second CPD component (blue) and the overlayed ADF line profile shown in grey colour. The EMCD-like component also shows such a tendency, but the correlation is weaker. (**g**) Spectral differences obtained before the subtraction of the second CPD component and (**h**) after the subtraction. Note how the subtraction of the second CPD component leads to a higher number of spectra showing clear EMCD signature in (**h**). Spectral differences were calculated from scaled spectra, so that their average peak value at *L*_3_ edge equals to one. In **g** and **h**, the individual spectral differences were vertically offset by 0.02 and 0.015, respectively.

Having theoretically optimized measurement conditions we proceed to the experimental verification. Two samples were examined, a 20 and 30 nm-thick polycrystalline layer of bcc iron with lateral sizes of the grains of the order of 100 nm. A grain oriented in the desired 3-beam condition with **G**=(110) has been found and several spectrum images have been acquired. For the further analysis of the data it is advantageous to acquire data in rectangular regions with the short side parallel to the atomic planes and the long side perpendicular to them, see Fig. 1d. Scanning in the direction parallel to the atomic planes one can minimize the effects of sample drift, since only a few pixels (typically <10) are acquired per scan row. A representative ADF image is shown in Fig. 3a.

### Data analysis

Due to a very low signal-to-noise ratio in individual spectra, a careful data analysis approach is needed. Details of the noise-reduction procedure are described in the Methods section. Two regions of interest (ROI, Fig. 3c) with a beam shift of 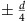, respectively, were determined on the basis of the ADF image. Summing all spectra within the region with expected positive/negative EMCD signals results in two spectra whose difference is expected to show an EMCD signal, as shown in Fig. 3g. Out of 22 acquired data sets in a given measurement session, five show an EMCD-like spectral signature. Although most of the other data sets lead to noisy structures of low amplitude, there are several cases strongly contrasting with the expected EMCD spectral shape, which have a similar amplitude as those five EMCD-like difference spectra. This naturally casts doubts about the validity of the APR-EMCD detection in this stage.

Further insight has been obtained by tensor decomposition techniques, specifically the canonical polyadic decomposition (CPD; see Methods section). CPD systematically reveals two components (see Fig. 3d) in every single acquired data set. One of them has a clear EMCD-like spectral signature while the other consists of two peaks located at edge positions, both having approximately the same height. Raw maps corresponding to the latter component show its localization on lattice planes, Fig. 3f, thus suggesting some sort of electron channelling effects. We suggest that this component should be explained by non-dipole transitions, because its presence requires transition matrix elements with a qualitatively different dependence on the momentum transfer vectors than the dominant dipole transitions. The EMCD-like component does not show any clear localization, its map seemingly being dominated by noise. However, subtraction of the second component as an additional pre-processing step in the data analysis described above increases the detection rate of EMCD-like spectra to over 50%, while it removes false positives, that is, difference spectra with non-EMCD spectral signature with comparable amplitude, Fig. 3h. See Supplementary Fig. 2, for a similar analysis for another measurement session, leading to the same conclusions.

Quantitative analysis of the results via sum rules^5^,^6^ is complicated due to the high noise level. Supplementary Fig. 3 summarizes the cumulative sums of the difference spectra^13^ shown in Fig. 3 and Supplementary Fig. 2. Except for the six noisiest curves showing an erratic behaviour in the pre- and post-edge region of their cumulative sums, preventing the application of sum rules, the orbital to spin moment ratios^5^,^6^ *m*_*L*_/*m*_*S*_ range between −0.1 and 0.3 with a mean value of 0.057 and a standard error of 0.030 (s.d. 0.120). The median value for the reduced and the whole data set is 0.050 and 0.072, respectively. Within an error margin given by the standard error, these values are in agreement with expected values for bulk iron^13^, supporting the interpretation of the extracted spectral differences as EMCD. We note that even though a few data sets lead to expected *m*_*L*_/*m*_*S*_ ratios on their own, an overall higher quality of data is needed to systematically obtain reliable *m*_*L*_/*m*_*S*_ ratios from individual data sets.

## Discussion

We have successfully demonstrated the detection of EMCD signal in our data sets, at the expense of enhanced data processing. Nevertheless, the EMCD extraction could be greatly simplified by an efficient elimination of sample drift. Even small drifts can cause inclusion of spectra with negligible EMCD into the ROIs, thereby increasing the noise level and mixing in the channelling effects into spectral differences. We expect that these issues will be overcome by advanced drift correction algorithms and/or by rapid acquisitions of large data sets on instruments equipped with the needed functionality.

Importantly, our APR-EMCD measurement method does not require any modifications to the microscope hardware^15^ nor requires utilizing probe correctors in non-standard ways^20^, while it offers EMCD spectra at near-atomic resolution from an area as small as 0.5 nm^2^, a resolution that is likely to be even more enhanced in future experiments. We expect that its ease of implementation will greatly contribute to its proliferation and becoming the method of choice for magnetic measurements at the sub-nanometre scale.

## Methods

### Derivation of the EMCD strength in APR-EMCD set-up

For a thin sample, the inelastic scattering cross-section can be written as:


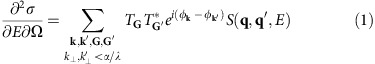


where **q**=**k**_*f*_−**k**−**G** and **q′**=**k**_*f*_−**k′**−**G′** and *S*(**q**, **q′**, *E*) is the mixed dynamical form factor^22^ (MDFF) describing intelastic transitions. For very thin samples *T*_**G**_ is ∼1 for **G**=0 and a small complex number for non-zero **G** vectors; *ϕ*_**k**_ describes the phase distribution in the profile of the probe. Summing over atoms in a crystal leads to a condition that **q**−**q′** and consequently also **k**−**k′** needs to be a reciprocal lattice vector. Neglecting elastic scattering entirely we obtain


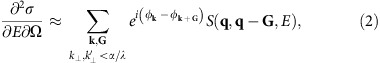


where **k′**=**k**+**G**. The magnetic signal is contained in the imaginary part of the MDFF (ref. 1) and the MDFF itself is Hermitian with respect to interchange of **q** and **q′**. Therefore we can write for the magnetic component of the scattering cross-section


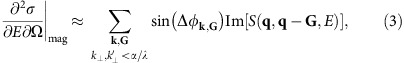


where Δ*ϕ*_**k,G**_=*ϕ*_**k**_−*ϕ*_**k**+**G**_. In the 3-beam geometry we can orient the *x*-direction along the discrete periodicity—perpendicular to lattice planes—leading to **G**=(*G*, 0, 0), which allows to express the imaginary part of the MDFF for magnetization along *z*-direction as


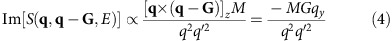


and the phase difference due to shifting the probe becomes Δ*ϕ*_**k,G**_=*G*Δ*x*. This shows that the EMCD signal oscillates as a function of displacement Δ*x* from the atomic planes with wavelength *d*, where *d* is the distance between planes, and its distribution in the diffraction plane is antisymmetric with respect to the *x* axis.

### Dynamical diffraction simulations

A multislice method was used to describe elastic propagation of the convergent electron beam through an orthogonal supercell of bcc iron of dimensions 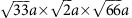, so that the *c* axis of this structure corresponds to propagation direction (1
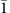
8) and the *b* axis is parallel to (110), perpendicular to the lattice planes. The spatial grid had a spacing of 0.068 Å in all three dimensions, and we considered a three-dimensional potential parametrized by Weickenmeier and Kohl^24^. Inelastic calculations were performed using the modified automatic term selection (MATS) algorithm, using the multislice results to describe the elastically scattered incoming convergent probe^23^. The convergence parameter in the MATS summation algorithm was set to 10^−6^.

### Sample preparation

20 and 30-nm-thick bcc iron layers covered by a 2-nm-thick Al cap layer (to prevent the oxidation of Fe) were deposited on 15-nm-thick Si_3_N_4_ membranes by thermal evaporation in an ultra-high vacuum molecular beam epitaxy system. The thicknesses were controlled using calibrated quartz microbalances. No *ex-situ* or *in-situ* preparation/cleaning was applied to the Si_3_N_4_ membranes before the deposition. The membranes were kept at room temperature during the deposition, but the Fe films were post-annealed at 750 °C for 120 min. before the deposition of the Al cap layers at room temperature. The disordered structure of the membranes (nanocrystalline or amorphous) results in a polycrystalline morphology of the metallic Fe/Al films. The post-annealing treatment led to a lateral grain size of the order 100 nm. Air exposure between sample growth and EMCD measurements oxidized the Al cap layer to a depth of ∼1.5 nm ensuring a closed AlO_*x*_ layer. Accordingly, no substantial oxidation of the Fe was observable in both electron energy loss spectra (EELS) and electron diffraction. Some metallic Al may remain at the interface to the iron film.

### Data acquisition

The measurements were carried out using EELS spectral imaging with an aberration-corrected scanning transmission electron microscope (STEM), the JEM ARM200F of the High-Voltage Electron Microscopy Laboratory, Nagoya University. The STEM was operated at 200 kV and equipped with a Gatan Image Filter Quantum. The beam convergence semi-angle was 10 mrad and the sample orientation was set to fulfil the symmetric 3-beam condition with **G**=(110), as shown in Fig. 1a, and a rectangular EELS entrance aperture was inserted as shown in Fig. 1d. The rectangular EELS aperture was implemented in the auxiliary slot next to the BF/ADF detectors of the Gatan Image Filter, which is conventionally used for a spatially-resolved EELS technique^25^. The spectrum image data sets were then obtained at a step width of 0.015 nm and an exposure of 0.2 s per spectrum, simultaneously collecting ADF images with the annular detector covering 50–90 mrad.

### Data analysis

In the first step of the analysis, the data sets were aligned on their energy axis using a cross-correlation procedure. The noise influence was reduced using robust principal component analysis (ROBPCA^26^) of the joint, aligned and mean-subtracted data set. Following the information of the scree plot, we reconstructed the data set using the first seven, most significant components. This estimate of the data dimension was also supported by the maximum likelihood estimator for the data dimension^27^. Subsequently, a power law background was subtracted from the data sets. Remaining sub-pixel instability of the *L*_2,3_ peak positions were corrected for by orthogonalizing the data set to the derivative of the mean spectrum. Omitting this step leads to EMCD-like differences in some cases, however, the derivative of the mean spectrum often dominates the difference spectrum. Such pre-processed data sets were used as an input for the analysis via spectral difference described in the next paragraph. For the CPD (see below), the mean spectrum has been subtracted after prior post-edge normalization.

ROIs were identified on the basis of ADF images, see Fig. 3b,c. A line profile of ADF image was smoothed for easier identification of the position of atomic planes. The local maxima of the line profile represent positions of atomic planes and local minima represent the region in the middle between two neighbouring planes. Then the ROIs have their centres in the midpoints between the neighbouring pairs of minima and maxima. The width of each ROI was chosen as 40% of the distance between its enclosing minimum and maximum. An example map of the ROIs is shown in Fig. 3c. Thus defined ROIs fall into two categories, being right or left from their closest atomic planes. Theory predicts opposite signs of EMCD contained in these two categories of ROIs. Consequently, two spectra are formed as averages of all individual spectra falling into these two groups of ROIs. Those two spectra are post-edge normalized and finally their difference is extracted, as shown in Fig. 3g.

Analysis via CPD (ref. 28) is performed as the last step of the data processing of the individual spectrum images, before extraction of the spectra using ROIs described in the previous paragraph. Its sole purpose is removal of the component shown in Fig. 3d in blue colour from the spectrum images. CPD provides a unique decomposition (in the mathematical sense), which is a desirable property. In CPD, each data point of a three-dimensional datacube, *p*_*k*,*l*,*m*_, is described as 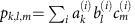 for a given number of components in a way that minimizes the sum of the squares of the residuals. Each of the terms in the sum is a datacube, where the information along each mode can be described by a single vector. Here, CPD is invoked for matricized datacubes and the third dimension is represented by the original data set with a small level of noise modelled as a uniformly distributed random variable. Before being subjected to the CPD, the resulting tensor was compressed using multilinear singular value decomposition. Due to the prior truncation via ROBPCA and further pretreatment, six components along spatial and energy modes suffice to capture the contained information. Two-component CPD leads reproducibly for all measured datacubes to the same two spectral components as shown in Fig. 3d.

### Data availability

All relevant data are available from authors.

## Additional information

**How to cite this article:** Rusz, J. *et al*. Magnetic measurements with atomic-plane resolution. *Nat. Commun.* 7:12672 doi: 10.1038/ncomms12672 (2016).

## Supplementary Material

Supplementary Information Supplementary Figures 1-3 and Supplementary References
