# Erratum: Magnetic measurements with atomic-plane resolution

**DOI:** 10.1038/ncomms13159

**Published:** 2016-10-05

**Authors:** Ján Rusz, Shunsuke Muto, Jakob Spiegelberg, Roman Adam, Kazuyoshi Tatsumi, Daniel E. Bürgler, Peter M. Oppeneer, Claus M. Schneider

Nature Communications 7: Article number: 1267210.1038/ncomms12672 (2016); Published: 08 31 2016; Updated: 10 05 2016

In the originally published version of the Article, an incorrect reference number was cited. The final sentence of the first paragraph of the Introduction section should read: ‘The likely reasons are technical difficulties in generating atomic size electron vortex beams, together with fine control of their resulting orbital angular momentum in the case of electron vortex beams, and a low EMCD signal fraction in the case of astigmatic beams^21^.'

