# Supplementary material for: Magnetic measurements with atomic-plane resolution
Source: Nat Commun. 2016 Aug 31;7:12672. doi: 10.1038/ncomms12672 (PMC5013673; doi:10.1038/ncomms12672)
Supplement: Supplementary Information — Supplementary Figures 1-3 and Supplementary References [file ncomms12672-s1.pdf]

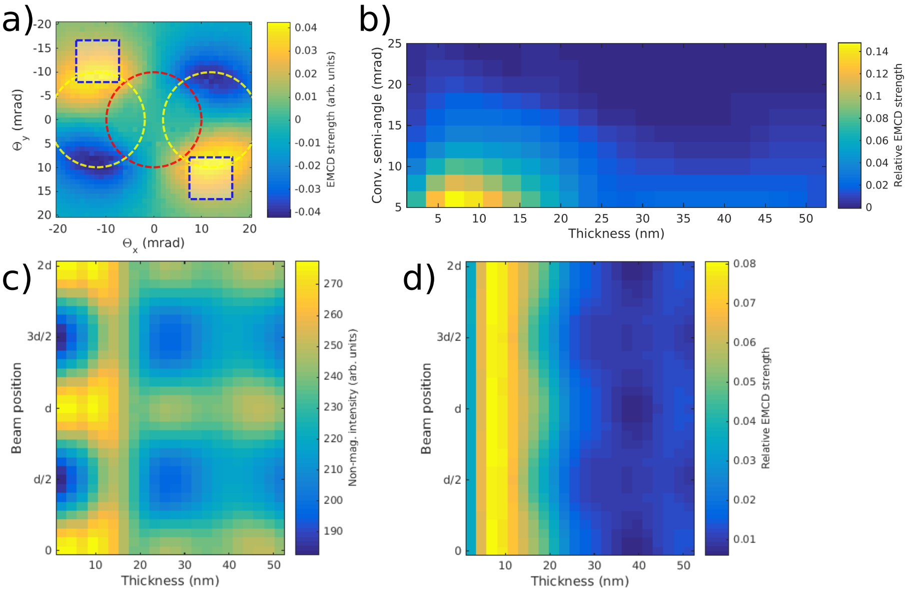

Supplementary Figure 1: **Simulations of classical EMCD in the three-beam geometry. a,** Example distribution of the Fe-L_3 edge EMCD signal in the diffraction plane for a beam centered on an atomic plane – here the contribution due to beam shift, described in the main text, vanishes regardless of the convergence angle. Positions of the transmitted beam disk (red circle) and Bragg scattered beam disks (yellow circles) are marked, as well as the rectangular detector region used for the evaluation of the magnetic signal. Note that the settings used here differ from the wide rectangular aperture described in the main text, which is used to detect EMCD originating from the beam shift (Fig. 1 of the main text). Positioning the collection apertures symmetrically on the minima (blue regions) would lead to an identical result, just with opposite sign of the EMCD signal. **b,** Optimization of the relative EMCD signal strength as a function of convergence angle and sample thickness. Note how the relative strength of the EMCD signal decreases as the convergence angle is increased. Yet, a sizable EMCD can be detected for thin samples with convergence angles of 15 mrad or even larger, suggesting that classical EMCD setups should be able to detect EMCD even in the atomic resolution regime. **c,** Calculated non-magnetic signal as a function of probe position and sample thickness at the Fe-L_3 edge and **d,** the corresponding relative EMCD strength. In **c** and **d,** the convergence semi-angle was set to 10 mrad. Note how the relative strength of EMCD signal only weakly depends on the beam position in between the planes. This is again in contrast with the EMCD signal obtained with the beam shift approach described in the main text.

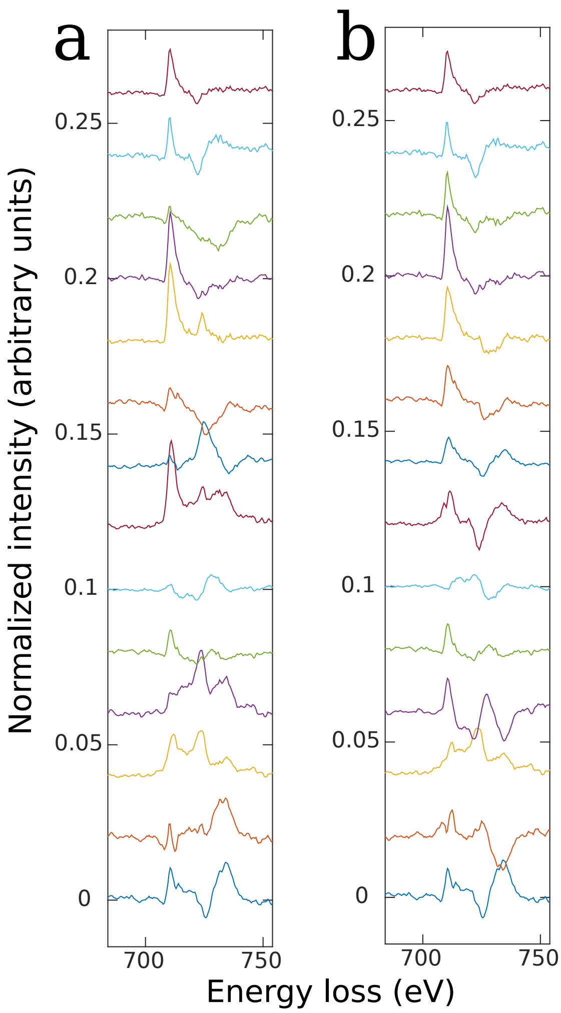

Supplementary Figure 2: **Experimental detection of EMCD with atomic plane resolution acquired in another measurement session. a,** Spectral differences obtained prior to the subtraction of the 2nd CPD component and **b,** after the subtraction. Note how the subtraction of the 2nd CPD component leads to a higher number of spectra showing clear EMCD signature in **b**. In this set of measurements a few spectra strongly deviating from the EMCD signature are still observed. Note that these spectra show no systematic shape, while still a majority of the datasets leads to spectral differences with clear EMCD signature. We also remind that, as in Fig. 3 of the main text, also here we do not do any sub-selection of the datasets. All acquired datacubes from the measurement session are presented.

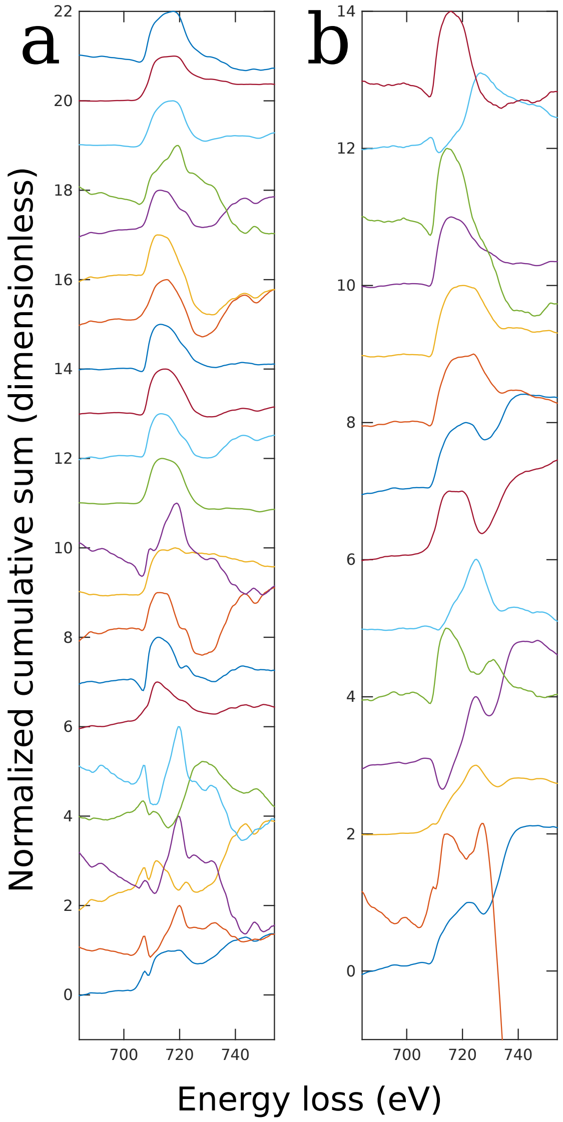

Supplementary Figure 3: **Cumulative sums of difference spectra: a,** for the dataset from Fig. 3 of the main text, after the subtraction of the 2nd CPD component, **b,** the same for the dataset from Supplementary Figure 2. The cumulative sums were normalized to 1 within the range of energies 708–723 eV, where the $L_{2,3}$ peaks are located. This allows to take $p = 1$ for the sum rules evaluation and q is extracted from the post-edge region as an average value from the energy range 730–750 eV. Then the m_L/m_S ratio is given by $m_L/m_S = 2q/(9-6q)$ [1, 2, 3]. A summary of the quantitative analysis for the dataset **a** is given in the main text. For the dataset **b,** eight of cumulative sums show a stable pre-edge and post-edge behavior, allowing to apply the sum rules. The mean m_L/m_S for this reduced dataset is 0.079, standard error and deviation are 0.053 and 0.15, respectively. The median of both reduced and complete datasets is equal to 0.089. Individual cumulative sums are vertically offset by 1.

Supplementary References

[1] Rusz, J., Eriksson, O., Novák, P. & Oppeneer, P. M. Sum rules for electron energy loss near edge spectra. *Phys. Rev. B* **76**, 060408(R) (2007).

[2] Calmels, L. *et al.* Experimental application of sum rules for electron energy loss magnetic chiral dichroism. *Phys. Rev. B* **76**, 060409(R) (2007).

[3] Muto, S. *et al.* Quantitative characterization of nanoscale polycrystalline magnets with electron magnetic circular dichroism. *Nature Comm.* **5**, 3138 (2014).
